# Perspectives for the Development of CD38-Specific Heavy Chain Antibodies as Therapeutics for Multiple Myeloma

**DOI:** 10.3389/fimmu.2018.02559

**Published:** 2018-11-06

**Authors:** Peter Bannas, Friedrich Koch-Nolte

**Affiliations:** ^1^Deptartment of Radiology, University Medical Center Hamburg-Eppendorf, Hamburg, Germany; ^2^Institute of Immunology University, Medical Center Hamburg-Eppendorf, Hamburg, Germany

**Keywords:** antibody engineering, CD38, heavy chain antibody, monoclonal antibody, multiple myeloma, nanobody

## Abstract

The NAD^+^-metabolizing ectoenzyme CD38 is an established therapeutic target in multiple myeloma. The CD38-specific monoclonal antibodies daratumumab and isatuximab show promising results in the clinic. Nanobodies correspond to the single variable domains (VHH) derived from heavy chain antibodies that naturally occur in camelids. VHHs display high solubility and excellent tissue penetration *in vivo*. We recently generated a panel of CD38-specific nanobodies, some of which block or enhance the enzymatic activity of CD38. Fusion of such a nanobody to the hinge, CH2, and CH3 domains of human IgG1 generates a chimeric llama/human hcAb of about half the size of a conventional moAb (75 vs. 150 kDa). Similarly, a fully human CD38-specific hcAb can be generated using a CD38-specific human VH3 instead of a CD38-specific camelid nanobody. Here we discuss the advantages and disadvantages of CD38-specific hcAbs vs. conventional moAbs and provide an outlook for the potential use of CD38-specific hcAbs as novel therapeutics for multiple myeloma.

## Introduction

CD38 is a cell surface ectoenzyme that metabolizes NAD^+^ released from damaged cells in inflammation (1). In concert with the ecto-enzymes CD203 and CD73, CD38 contributes to the conversion of NAD^+^ to immunosuppressive extracellular adenosine. In the tumor microenvironment, CD38 may promote tumor growth by suppressing effector T cell responses (1, 2). Since CD38 is overexpressed by multiple myeloma cells and other hematological tumors, it has attracted interest as a target for therapeutic antibodies (3–5).

Nanobodies are single domain antibody fragments derived from the heavy chain IgG antibodies naturally occurring in llamas and other camelids (6–8). In these animals, the IgG2 and IgG3 isotypes lack the CH1 domain and do not bind to light chains. Nanobodies correspond to the variable domain (VHH) of these heavy chain antibodies. VHHs carry characteristic residues in the framework region 2 (FR2) that render them highly soluble in the absence of a paired light chain (8–10). VHHs often have a long complementarity determining region 3 (CDR3) that can mediate binding to the catalytic cavity of an enzyme and other hidden epitopes that are not accessible for conventional antibodies (11–13). Their robust, soluble single domain format renders nanobodies amenable for genetic fusion to the hinge and Fc domains of other antibody isotypes (14, 15). Owing to their high solubility, it is much easier to link two or more VHHs into bi- or multivalent formats than the corresponding VH+VL domains of conventional antibodies.

## CD38-specific therapeutic chimeric mouse/human and fully human conventional antibodies

**Figure 1:**
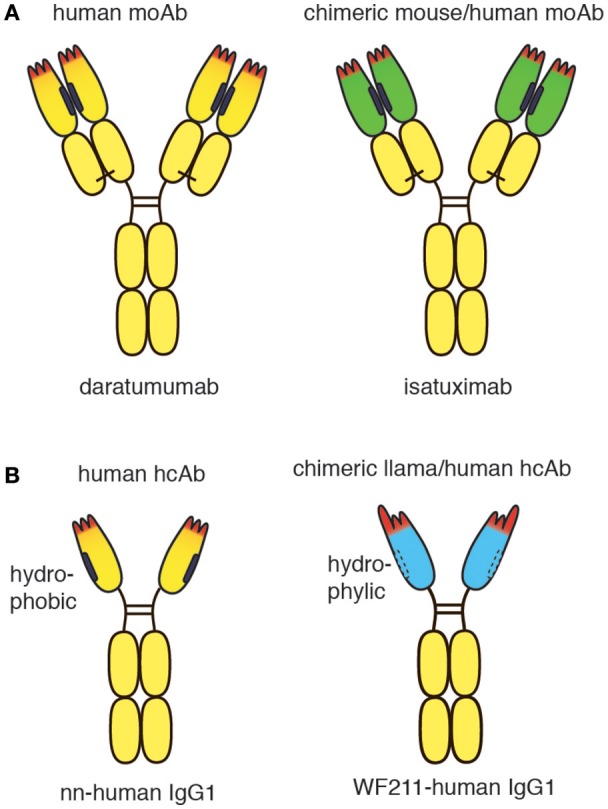
Schematic diagram of conventional and heavy chain CD38-specific antibodies. **(A)** Schematic diagrams of therapeutic CD38-specific conventional antibodies. Daratumumab is derived from a CD38-immunized human-antibody transgenic mouse. Isatuximab is derived from a CD38-immunized wildtype mouse. The chimeric antibody was generated by genetic fusion of the VH and VL domains of the mouse monoclonal antibody to the constant domains of human IgG1 and kappa, respectively. **(B)** Schematic diagrams of CD38-specific heavy chain antibodies. A fully human CD38-specific heavy chain antibody has been derived from a CD38-immunized human heavy chain transgenic rat. The chimeric WF211-human IgG1 heavy chain antibody was generated by genetic fusion of the VHH domain of a llama heavy chain antibody to the hinge, CH2 and CH3 domains of human IgG1. Human heavy chain antibodies display a natural “stickiness,” i.e., tendency to bind light chains via the hydrophobic interface that helps to stabilize the orientation of the VH-VL pair in conventional antibodies. In contrast, chimeric llama-human heavy chain antibodies do not show any natural “stickiness” or tendency to bind light chains. VHH domains have been shaped by 50 Mio years of evolution for high solubility in the absence of a light chain.

The conventional CD38-specific moAbs daratumumab and isatuximab have proven therapeutic efficacy in multiple myeloma (5, 16). Both antibodies were derived from mice immunized with human CD38. While daratumumab was generated from CD38-immunized transgenic mice that carry genomic loci encoding human IgH and IgL (17), isatuximab was generated from CD38-immunized wild type mice (18). The VH and VL domains of the murine moAb were genetically fused to the CH1-hinge-CH2-CH3 domains of human IgG1 and to the constant domain of the kappa light chain (Cκ), respectively, generating a classic mouse/human chimeric antibody (Figure 1A). The crystal structure of isatuximab in complex with CD38 indicates that its capacity to inhibit the enzymatic activity of CD38 is by an allosteric mechanism (18). Recently, the VH and VL domains of daratumumab were used to construct a single-chain human anti-CD38 cytokine-antibody fusion protein termed IL2-αCD38-αCD38-scTRAIL (19). The bivalent tandem scFv of daratumumab mediated specific binding to CD38 expressing myeloma cells, while the engineered homotrimeric format of TRAIL induced apoptosis of these cells, presumably by binding to cognate death receptors.

## CD38-specific chimeric llama/human and fully human heavy chain antibodies

Recently, CD38-specific nanobodies were generated from CD38-immunized llamas (20, 21). Some of these nanobodies inhibited or enhanced CD38 enzymatic activity in a dose dependent manner and effectively targeted CD38 on human tumor cells in a mouse Xenograft model (20). Several nanobodies bind independently of daratumumab. Such nanobodies have proven useful for detecting cell surface CD38 in patients treated with daratumumab (22). Owing to their high solubility, the nanobodies can readily be fused to other protein domains, including the hinge and Fc domains of human IgG1 (Figure 1B). Such chimeric llama/human heavy chain antibodies acquire the capacity to induce classic Fc-mediated effector functions, including ADCC and CDC (23).

In contrast to the hydrophylic nature of camelid VHH domains, human VH domains display a natural “stickiness” and tendency to aggregate in the absence of a light chain (24–26). This “stickiness” is attributed to the hydrophobic interface that helps to properly orient VH and VL domains for joint interaction with the target antigen (27). “Camelization” of human VH domains by substituting hydrophobic amino acid residues in FR2 with hydrophilic residues can greatly enhance the solubility of human VH domains (28–30). In order to express human heavy chain antibodies in transgenic mice or rats it is therefore advisable to inactivate not only the endogenous rodent heavy chain locus, but also the kappa and lambda light chain loci. Interestingly, during the ensuing immune response, somatic hypermutation and selection drive the expansion of VH variants that increase the solubility of heavy chain antibodies (31, 32). A similar mechanism was observed when human VH domains were affinity matured *in vitro* (33). Recently, CD38-specific human heavy chain antibodies were generated successfully from CD38-immunized human heavy chain-only transgenic rats (32).

## Advantages and disadvantages of CD38-specific heavy chain antibodies vs. conventional moAbs

### Tissue penetration and *in vivo* half life

Heavy chain antibodies are only half the size of conventional moAbs (75 vs. 150 kDa). HcAbs may therefore penetrate more effectively into CD38-expressing tumors than full size moAbs, particularly when the tumors promote increased interstitial pressure. Better tissue penetration has indeed been demonstrated for nanobodies vs. conventional antibodies in solid tumors and subcutaneous tumors (34, 35). Tissue penetration is a highly relevant issue, in particular when considering that multiple myeloma resides in the bone marrow and is surrounded by a dense immune suppressive microenvironment (1). It will be important to determine whether nanobody-based hcAbs do reach myeloma cells in the bone marrow more efficiently than conventional antibodies.

The half life of therapeutic antibodies is influenced by several factors, including size, glycosylation, and affinity to the neonatal Fc receptor. While a smaller size may facilitate tissue penetration, a small size may also facilitate renal filtration and thereby shorten the persistence of the therapeutic *in vivo*. Several strategies have been employed successfully to prolong the *in vivo* half life of nanobodies, including conjugation to polyethylene glycol polymers (36), genetic fusion to an albumin-specific nanobody (34, 37). In case of nanobody based hcAbs, Fc engineering could be used to introduce mutations that enhance binding to the neonatal Fc receptor and thereby prolong persistence *in vivo* (38, 39).

### Developability of bispecific therapeutics

The soluble nature of the nanobody VHH domain, facilitates the construction and production of bispecific antibodies. For example, a bispecific nanobody-based heavy chain antibody can readily be generated simply by fusing a second nanobody to the N-terminus of a nanobody-based hcAb. Importantly, nanobody-based bispecific hcAbs are composed of two identical polypeptide chains, i.e., their production does not require any “knob in hole” technology or adjusting the of expression levels of two or more vectors (40, 41). This simplifies the production and developability of bispecific hcAbs, although the moderate increase in size of a bispecific vs. a mono-specific hcAb (from ~75 to ~100 kDa) may compromise tissue penetration. By tandem fusion of two nanobodies that recognize independent epitopes of CD38 to the Fc domain of human IgG, we recently generated tetravalent biparatopic hcAbs that exhibit a markedly enhanced capacity to induce CDC of CD38-expressing myeloma cells.

### Modulation of enzyme activity

Owing to the inherent capacity of nanobodies to extend into and block active site crevices (11, 12), a heavy chain antibody containing a CD38-antagonistic nanobody may provide an additional therapeutic benefit by inhibiting the production of immunosuppressive adenosine (1, 2). Conceivably, the potency of enzyme inhibition may be enhanced by fusion of an enzyme-inhibiting nanobody to a nanobody recognizing a distinct epitope of CD38, e.g., in a biparatopic activity blocking hcAb.

### Immunogenicity

The potential immunogenicity of antibody therapeutics is a relevant concern (5, 42, 43). The development of neutralizing antibodies against the therapeutic antibody by the patient usually renders the patient resistant to the therapeutic. This risk for developing such antibodies is larger for chimeric antibodies that contain murine VH and VL domains such as rituximab and isatuximab than for fully human antibodies such as dartumumab which is composed only of human domains. However, it is impossible to fully humanize the idiotype of an antibody without losing specificity of effectivity since the unique CDR loops of the VH and VL domains are required for specificity. Hence, the potential development of antibodies directed against the unique CDR loops remains a concern for any therapeutic antibody. Drug antibodies have not yet been detected in any daratumumab-treated patients (5). However, it is uncertain to what extent this is due to the lack of a sensitive assay for such antibodies.

**Figure 2:**
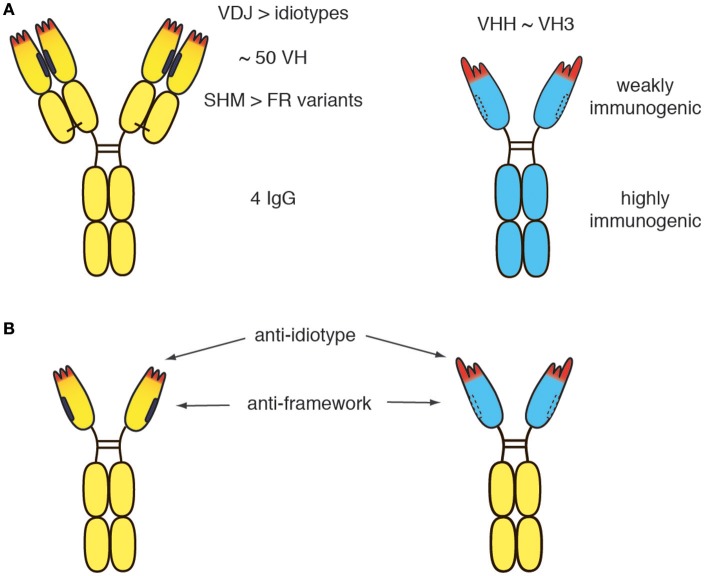
Potential immunogenicity of heavy chain antibodies. **(A)** The human germline encodes ~50 distinct VH domains and 4 distinct IgG isotpyes. V-D-J recombination during B-cell development generates millions of distinct idiotypes (antigen binding paratopes, CDR regions 1, 2 indicated in red). Subsequent to antigen encounter, somatic hypermutation generates many more variant VH domains. During pregnancy, maternal IgGs are translocated through the placental trophoblasts to the fetus, leading to tolerization of the new born human immune system against millions of VH variants, but only 4 distinct IgG isotypes. **(B)** In germline configuration, llama VHH domains show ~80-90% amino acid sequence identitiy to human VH3 domains. A few amino acid substitutions in the VL face (mainly framework region 2, indicated by dashed lines) and a long CDR3 that can partially fold back onto this face largely account for the dramatically improved solubility of camelid VHH domains *vs*. human VH3 domains. The solubility of human VH can be improved by “camelization,” i.e., by replacing hydrophobic residues at the interface of the VL domain (indicated in black) with hydrophylic residues resembling those found in VHH domains. Conversely, camelid VHH domains can be “humanized,” i.e., by replacing amino acid residues in the framework with residues corresponding to germlin human VH domains. However, the idiotype of a therapeutic moAb or hcAb cannot be fully humanized without compromising binding to the target antigen. Similarly, the VL face cannot be fully humanized without compromising solubility. Therefore, small risks remain, that the patient will develop antibodies against the idiotype and/or against the (much smaller) hydrophilic VL face.

The human germline encodes ~50 distinct VH domains and 4 distinct IgG isotypes (Figure 2A) (44, 45). V-D-J recombination during B-cell development generates millions of distinct idiotypes (antigen binding paratopes). Subsequent to antigen encounter, somatic hypermutation generates many more variant VH domains. During pregnancy, maternal IgG is translocated from the maternal blood through placental trophoblasts into the blood stream of the fetus, leading to tolerization of the new born immune system against millions of VH variants, but only 4 distinct IgG isotypes. In germline configuration, llama VHH domains show ~80–90% amino acid sequence identity to human VH3 domains, i.e., the predominant VH subset found in human immunoglobulins (46). As a result of somatic hypermutation, two matured human VH domains often differ more from one another than a germline human VH3 domain from a llama VHH3 domain. A few hydrophilic amino acid residues in framework region 2 and the long CDR3 that can partially fold back onto the former interface to the VL domain largely account for the dramatically improved solubility of camelid VHH domains vs. human VH3 domains. These residues cannot be fully humanized without compromising solubility. Notwithstanding, the idiotype (CDR regions 1, 2, and 3) cover a much larger space (both, in the literal sense and in terms of potential immunogenicity) than these hydrophilic amino acids in the former VL interface.

Although the immunogenicity of a therapeutic antibody can be reduced by humanization, the residual risk remains for any therapeutic antibody that the patient develops antibodies directed against the idiotype (Figure 2B). Such anti-drug antibodies usually render the therapeutic useless for the patient. If more than one therapeutic antibody is available for a particular target, an option in such cases is to switch to a different biologic targeting the same molecule (e.g., from daratumumab to isatuximab or vice versa). It is conceivable that in the future, the risk of developing anti-drug antibodies can be reduced further by tolerization strategies that will become available and permit tolerization of the patient to the therapeutic antibody before treatment is initiated.

## Conclusions and outlook

Later this year, Caplacizumab, a dimeric nanobody directed against the van Willebrand factor, is expected to receive FDA approval as the first nanobody in the clinic (47, 48). Nanobodies that antagonize CD38 provide proof of concept for the notion that these small biologics represent attractive alternatives to small molecule inhibitors for inhibiting the production of immunosuppressive adenosine. Nanobody-based heavy chain antibodies retain all effector functions of full sized moAbs, at half the size. This size advantage will likely facilitate targeting of tumor cells *in vivo*, even under conditions of increased interstitial pressure within tumors. Owing to their excellent solubility, it is much easier to link different nanobodies in a single therapeutic than the combined VH+VL domains of conventional moAbs. The high solubility of CD38-specific heavy chain antibodies may come at the price of a slightly higher risk for inducing anti-drug antibodies compared to conventional human CD38-specific moAbs. In addition to the complementarity determining regions, the VL face of heavy chain antibodies may provide a second, albeit much smaller, vulnerability than the idiotype. It will be interesting to see whether “humanized” nanobody heavy chain antibodies or “camelized” human heavy chain antibodies will hold the leading nose in the race to the clinic.

## Author contributions

PB and FK-N conceived the project. FK-N wrote the manuscript. Both authors reviewed and approved the manuscript.

### Conflict of interest statement

FK-N receives a share of antibody sales via MediGate GmbH, a wholly owned subsidiary of the University Medical Center Hamburg-Eppendorf. PB and FK-N are co-inventors on a patent application on CD38-specific nanobodies. The Handling Editor declared a past co-authorship with one of the authors, FK-N.

## Abbreviations

ADCC: antibody-dependent cytotoxicity
CDC: complement-dependent cytotoxicity
CDR: compelementarity determining region
Fc: crystallizing fragment
FR: framework region
Ig: immunoglobulin
kD: kilodalton
NAD: nicotinamide adenine dinucleotide
moAb: monoclonal antibody
Nb: nanobody
Nb-hcAb: nanobody-based (human) heavy chain antibody
scFv: single chain variable fragment
VH: variable domain of a conventional heavy chain
VHH: variable domain of a camelid heavy chain antibody.
